# How Successful is Combined Superior and Inferior Oblique Muscle Surgery in Young Children with Superior Oblique Underaction Presenting in Infancy with a Severe Head Tilt?

**DOI:** 10.22599/bioj.171

**Published:** 2021-02-11

**Authors:** Revelle A. Littlewood, John P. Burke

**Affiliations:** 1 Sheffield Teaching Hospitals NHSFT, GB

**Keywords:** Congenital superior oblique palsy, Torticollis, Vertical diplopia

## Abstract

**Background/Objective:**

To evaluate the success of combining ipsilateral inferior and superior oblique muscle surgery in young children with congenital unilateral superior oblique under action who present in infancy with a large socially noticeable head-tilt.

**Methods:**

A consecutive retrospective case series of young children was analysed. The success of surgery in eliminating the head-tilt was evaluated by pre- and post-operative ocular motility assessment focusing on the vertical misalignment in primary position and downgaze, the magnitude of the head-tilt in degrees and the status of the superior oblique tendon.

**Results:**

Five children had a mean age at first surgery of 41 (range 25–63) months, a mean primary position vertical deviation of 26 (25–30) prism dioptres, a head-tilt of 30 (20–35) degrees and a mean post-operative follow up of 24 (8–43) months. While there was a uniform surgical plan, nonetheless each operation required individualisation based on a spectrum of per-operative superior oblique tendon findings. The head tilt was eliminated in 40% and reduced in the remainder, to a mean of 7 (0–18) degrees and with a mean post-operative primary position vertical misalignment of 3 (range 0–10) and of 10 (range 0–40) prism dioptres in downgaze.

**Conclusion:**

Combined, ipsilateral oblique muscle surgery reduced the severe head tilt and primary position alignment to a psychosocially and functionally acceptable level. For the majority, the outcome was stable or associated with further decremental improvement. A persistent downgaze vertical tropia occurred in children with macroscopically abnormal superior oblique tendons but these cases were not identifiable clinically pre-operatively.

## Introduction

Congenital or early onset torticollis can result from both orthopaedic and ophthalmic related pathologies, and when prominent, can be disabling for a young child and distressing for parents. Unilateral, superior oblique muscle underaction (SOUA)/inferior oblique overaction (IOOA) is the commonest cause of vertical strabismus associated with a compensatory head-tilt (CHP) or torticollis, where the child assumes excessive lateral or side-flexion of the cervical spine towards the shoulder, the normal range being up to 45 degrees (Corrigan & Maitland 1983) with/without other malposition’s including face-turn and/or chin elevation/depression.

Overtime, untreated CHPs’ lead to musculoskeletal sequelae that includes scoliosis and tight sternocleidomastoid (SCM) muscles, where SCM tightness can explain or contribute to residual torticollis following successful strabismus surgery (Lau et al. 2009). Expediting strabismus surgery before chronic musculoskeletal sequelae develop/progress is a goal. There exists experimental concern relating to the neurotoxicity risk of general anaesthesia in young children, and although the risks are not fully understood (Clausen, Hansen & Disma 2019; Rappaport et al. 2015), it appears prudent where practical to minimise the frequency of elective procedures in this age-group.

Patients, who present under the age of 2 years with SOUA/IOOA and a very prominent head-tilt, account for a numerically uncommon but socially obvious subgroup. Many more present as older children and adults with diplopia where torticollis is not the presenting symptom. Anecdotally, this frequently studied older group have more successful surgical outcomes than those presenting as infants/toddlers with severe torticollis. The prognosis for the functional and psychosocially prominent head-tilt (CHP) amongst this infant/toddler sub-group is not well documented in the literature. Two small studies that included such children reported that multiple extra-ocular muscle surgeries can be necessary to avoid residual symptomatic torticollis (Ahn et al. 2012; Lau et al. 2009). Lau et al (2009) and subsequently Ahn et al (2012) described children whose mean age at surgery was 6.8 (range 2.3–18) years and 10.1 (range 1.6–34) years, respectively, with large primary position (PP) vertical deviations with a mean of 14.3 (range 9–30) prism dioptres (PDs), and 12.4 ± 6.3 PDs respectively where 70% and 75% had successful treatment of torticollis. Neither study described superior oblique traction test (SOTT) findings or involved surgery on the superior oblique muscle.

Kekunnaya and Isenberg (2014) treated a cohort of 11, primarily young children aged 7 months to 27 years, whose mean age at surgery when the 27 year old was excluded was 3.1 years with a mean primary position hypertropia (HT) of 19 (range 6–30) PDs and a head-tilt of 17 (range 10–40) degrees with combined inferior oblique recession and superior oblique tuck nasal to the superior rectus muscle, irrespective of the deviation and torticollis size, with a success rate of 62%. All superior oblique tendons (SOT) were lax on traction testing while their macroscopic appearance at surgery was not described, nor was the post-operative alignment in the reading or downgaze positions where the deviation can be at its largest. Following additional sequential vertical muscle recession in 38% of their cases, the primary position hypertropia and torticollis was successfully treated in 100% (i.e., HT of 2 ± 6 PDs, head-tilt of 2 ± 2 degrees).

In this case-series, we evaluated the relative laxity and macroscopic appearance of each affected SOT and the limitations and effect of ipsilateral combined surgery on the superior and inferior oblique muscles as a single or sequential operative procedure in infants/toddlers with severe torticollis, large incomitant vertical deviations and fundus excyclotorsion.

## Subjects and Methods

Study approval of established surgical practice was granted in line with Sheffield Children’s Hospital protocols. A retrospective, consecutive case series from 2015–2019 of five children referred to a secondary and tertiary regional adult/paediatric strabismus unit for management of severe torticollis associated with previously diagnosed or suspected congenital SOUA was performed. All parents historically described the presence of torticollis in infancy and a subsequent or simultaneous awareness of a vertical strabismus. Previous strabismus surgery details were obtained from medical records.

**Figure 1:**
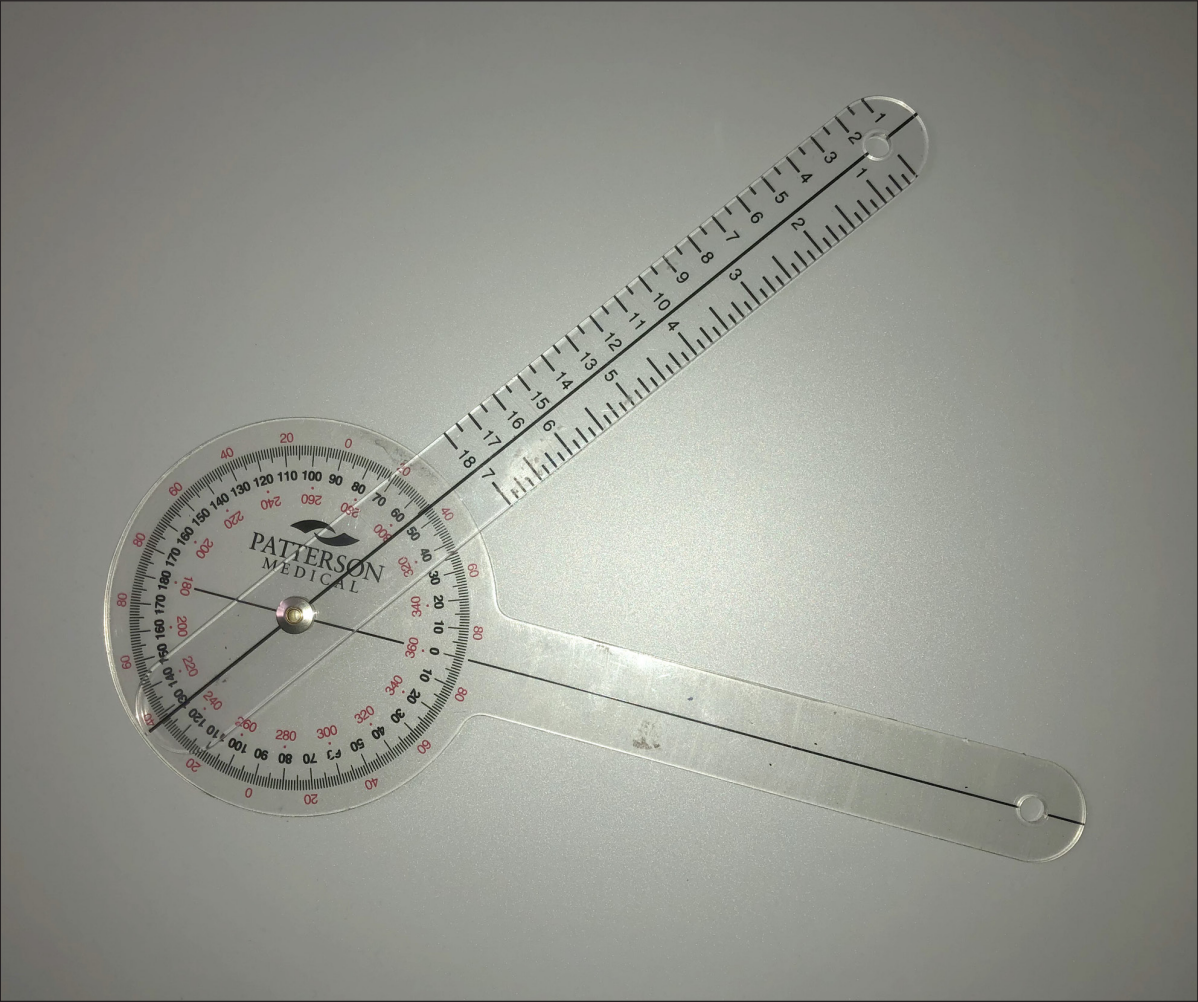
Goniometer.

Orthoptic evaluation consisted of an age-appropriate method of obtaining visual acuity. Pre- and post-operative ocular motility assessment included a binocular single vision (BSV) assessment, cover test, alternate cover and prism test with and without a CHP in primary position and without a CHP in eccentric gaze positions, as individual cooperation allowed from one consultation to another, until a diagnosis and a surgical management plan was formulated. Extra-ocular muscle under and over actions were recorded on a 0–4 scale (Vivian & Morris 1993). A graded hand-held Goniometer (Patterson Medical UK, ***Figure 1***) was used pre- and post-operatively to document the magnitude of the head-tilt in degrees while the child fixated on a target at eye level, with one arm of the goniometer placed perpendicular to the floor and the other parallel to the axis of the head tilt. Any additional face turn and/or abnormal chin position was documented but not measured objectively. The degree of head-tilt was graded as mild (<10 degrees), moderate (10–20 degrees) and severe (>20 degrees). All 5 cases had dilated pupil binocular indirect ophthalmoscopy (BIO) using a 20 dioptre Volk lens pre-operatively. Dilated fundus BIO was not routinely performed post-operatively. Behavioural immaturity precluded objective synoptophore evaluation of torsion.

The pre-operative findings and the post-operative findings from the latest follow-up were used for analysis (***Tables 1*** and ***2***).

**Table 1:** Age at and duration of follow-up after surgery, operative procedure, traction test and cyclotorsion findings.


PATIENT NUMBER	DIAGNOSIS	PREVIOUS SURGERY	FUNDAL EXCYCLOTORSION	INTRA-OPERATIVE SOTT	SURGERY	POST-OPERATIVE FOLLOW UP (MONTHS)

1	Right SOP	Right IO myectomy (2 years, 5 months)	Yes Right	Right SO – very lax	Right SO resection and advancement (3 years, 7 months)	38

2	Right SOP	Right IO myectomy (5 years, 3 months)	Yes Right	Right SO – very lax	Right SO resection and advancement (6 years, 6 months)	43

3	Left SOP	N/A	Yes Left	Left SO – moderately lax	Left SO Harada-Ito and Left IO recession (5 years, 3 months)	24

4	Right SOP	N/A	Yes Right	Right SO – moderately lax	Right Harada-Ito plus and Right IO myectomy (2 years, 2 months)	8

5	Right SOP	Right IO recession (3 years, 1 month)	Yes Right	Right SO – very lax	Right SO resection & advancement and Right IO myectomy (5 years, 2 months)	9


SOP – superior oblique palsy, IO – inferior oblique, SO – superior oblique, N/A – not applicable, SOTT – superior oblique traction test.

**Table 2:** Pre- and post-surgery head-tilt, oblique muscles versions, vertical misalignment in prism dioptres.


PATIENT NUMBER	PRE-OPERATIVE HEAD TILT IN DEGREES*	PRE-OPERATIVE OBLIQUE VERSIONS	PRE-OPERATIVE VERTICAL DEVIATION IN PDS, IN FORCED PRIMARY-POSITION	POST-OPERATIVE HEAD TILT IN DEGREES*	POST-OPERATIVE OBLIQUE VERSION ACTIONS	POST-OPERATIVE VERTICAL DEVIATIONS IN PDS

1	Severe left 35	Right SO –3 Right IO +2	PP: 30RHT DG: n/a	Mild left 7	Right SO –1 Right IO +1	PP: 0 DG: 5RHT

2	Severe left 32	Right SO –4 Right IO +1	PP: 30RHT DG: 40RHT	Moderate left 10 (18)	Right SO –3 Right IO +1	PP: 10RH(T) DG: 40RHT

3	Severe right 35	Left SO –3 Left IO +3	PP: 25LHT DG: 25LHT	Mild right 5	Left SO –1 Left IO 0	PP: 4LH DG: 4LH

4	Moderate left 20	Right SO –4 Right IO +4	PP: 25RHT DG: n/a	Mild left 5	Right SO –2 Right IO 0	PP: 1RH DG: 1RH

5	Severe left 30	Right SO –3 Right IO +3	PP: 25RHT DG: 30RHT	No tilt 0	Right SO –1/–2 Right IO 0	PP: 1RH DG: 4RH(T)


SO – superior oblique muscle, IO – inferior oblique muscle, PDs – prism dioptres, PP – primary position, DG – downgaze, HT – hypertropia, H – hyperphoria, H(T) – intermittent hypertropia, n/a – not documented, * Head posture grading, mild < 10 degrees, moderate 10–20 degrees, severe > 20 degrees.

Surgery: Each superior oblique tendon was isolated via a supero-temporal fornix incision. Its laxity and its macroscopic appearance were assessed and then each operation was individualised, where the aim was to improve the tone of the tendon on the basis of achieving normal or mild tightness on superior oblique traction testing (SOTT) (Guyton 1981), where possible at the completion of surgery.

All five superior oblique tendons were reinserted directly onto the sclera, using 6.0 vicryl except for case 2, where a non-absorbable 5.0 dacron was selected. The resected/transposed fibres of each SO tendon (SOT) were re-sutured onto sclera 8mm posterior to the superior border of the lateral rectus with the more posterior fibres positioned more posteriorly adjacent and parallel to the lateral rectus muscle belly.

The goal of an inferior oblique muscle myectomy or recession was to achieve a lax inferior oblique traction test (IOTT) post-operatively.

## Results

The mean age at the first operation for all children was 43.6 (range 26–63) months and at the initial/subsequent procedure was 54.4 (range 26–78) months. The follow up period after the last procedure was a mean of 24 (range 8–43) months (***Table 1***).

The maximum mean head-tilts in degrees pre-operatively was 30 (range 20–35) degrees and at the last post-operative visit, a mean of 7 (range 0–18) degrees (***Table 2***).

The mean primary position vertical deviation without a CHP was 26 (range 25–30) PDs. The mean downgaze vertical deviation, owing to behavioural immaturity was reliably documented in 3 cases and the mean deviation size was 32 (range 25–40) PDs. At the last follow-up visit the mean primary position vertical deviation was 3 (range 0–10) PDs while the mean downgaze deviation in all 5 cases was 10 (range 0–40) PDs (***Table 2***).

Clinically, all patients had subnormal BSV and/or stereoacuity documented pre-operatively whilst adopting a prominent CHP, and this status persisted post-surgery in primary position with a much reduced or resolved CHP.

All cases had confirmed unilateral fundus excyclotorsion consistent with their preferred CHP pre-operatively. Dilated fundus BIO was not routinely performed post-operatively. All cases had pre- and post-operative measurement of the head-tilt with a hand-held goniometer.

Prior surgery details from the referring ophthalmologist indicated that 3 children had either a primary inferior oblique myectomy (IOM) (Cases 1 & 2) or a primary inferior oblique recession (IOR) (Case 5). Parents historically described some (case 5) to no (cases 1 & 2) improvement in the torticollis in the immediate post-operative period but described an immediate, more obvious temporary reduction in the HT. The primary position hypertropia (ppHT) pre-operatively and between 1 to 4 weeks post-operatively in Cases 1 and 2, respectively, had measured 30 and 25 PDs pre-surgery and 25 and 14 PDs post-surgery respectively, whilst case 5 measured 18 PDs right HT at 6 months post-primary surgery.

We performed sequential superior oblique resection/transposition in two (cases 1 & 2) children and combined superior (SO) and inferior (IO) oblique muscle surgery in three—primary in cases 3 & 4, and an IO revision/myectomy in case 5 (Squirrell, Sears & Burke 2007). All 5 superior oblique tendons (SOTs’) were identified at or just temporal to the temporal pole of the superior rectus muscle. The SOT tone and morphology were variably abnormal such that the anterior half of the SO was transposed in three children (cases 3, 4 & 5), while the whole tendon was transposed in the other two (cases 1 & 2). All transposed fibres were resected prior to transposition because of varying levels of SOT laxity from marked to extreme (resection range 3–12mms’) (Helveston 1993; Helveston et al. 1996; Wheeler 1934).

We noted 2 patterns of SOT abnormality. One group (group A; cases 1, 2 & 5) demonstrated marked tendon laxity on SOTT and macroscopically anomalous tendons, incorporating considerable non-tendinous soft tissue. As these tendons were so lax, all underwent a Harada-Ito plus procedure where all of the SOT was transposed anterolaterally and combined with a 12mm and 5mm SOT resection in cases 1 & 2, respectively, while case 5 underwent a 4 mm resection of the anterior 50%+ of the SOT. The aim of SO surgery was to have a normal to slightly tight SOT at the end of the operation but this was not achieved in two cases (case 2 & 5) due to their macroscopically abnormal consistency.

Children in group B (cases 3 & 4) had moderate SOTT laxity and a macroscopically normal SOT appearance. The post-operative SOTT in this group demonstrated normal to a slightly increased SOTT tone.

It had not proved possible to distinguish between these patients categorised into groups A and B by ocular motility features alone. Post-operatively all group A cases demonstrated a residual incomitant hypertropia (HT) in downgaze, this being notable in case 2. This was not a feature of cases in group B.

In the early post-operative period, all cases had a mild to moderate Browns pattern of –1 to –3 under action that resolved over 3 months, with the exception of case 4, who has a –1 asymptomatic Browns at 8 months.

With the exception of case 2, the initial improved post-operative alignment, torticollis and BSV either remained stable or improved during follow-up (***Tables 1*** and ***2***). With case 2, the initial improved post-operative alignment and torticollis regressed over 6 months, particularly in downgaze, but then remained stable longer-term with BSV, including stereovision (Frisby stereoacuity = 55arc/second) in primary position, stable variable head-tilt that was maximal (10–18 degrees) when reading in primary position, and vertical diplopia was avoided in downgaze by monocular viewing.

## Discussion

The surgical management of the severe torticollis that manifested itself in infancy in this report represents an uncommon, well recognised subset of congenital USOUA whose outcome is not well reported in the literature. In this series, parents historically described two patterns of presentation. Three (60%) children presented initially with obvious torticollis prior to their subsequent awareness of vertical strabismus, while in the other group (cases 4 & 5), parents describe the simultaneous appearance of torticollis and strabismus in infancy that progressed prior to ophthalmology referral.

Variable co-operation in very young children is a potential limitation to making a timely diagnosis and surgical plan. There are no universally accepted minimal diagnostic criteria, but a complete motility examination incorporating nine gaze positions and head-tilts is not pragmatic. The assessment can be opportunistic, tailored and on occasions ‘piecemeal’ so as to mitigate against a breakdown in clinical co-operation with toddlers in particular, since professional or parental neutralisation of the CHP can lead to a sometimes persistent and unwanted change in the child’s behaviour. Lee et al (2018) demonstrated that a combination of steps 1 and 3 of the Parks three-step test was more sensitive than all three steps in diagnosing superior oblique palsy. All five children in this series had a large primary-position hypertropia (HT), which increased on both contralateral gaze and ipsilateral head-tilt and was associated with ipsilateral fundus excyclotorsion. These findings, in conjunction with the clinical history, were considered diagnostic for corrective strabismus surgery.

Historically, unilateral superior oblique underaction is classified by the magnitude of the primary position vertical deviation allied to specific patterns of vertical misalignment in eccentric gazes; and by an anatomical classification, in non-acquired cases that describe 4 categories of anomalous SOT, while magnetic resonance imaging (MRI) characteristics have added to our clinical and surgical understanding (Helveston et al. 1992; Knapp 1974; Scott & Kraft 1986). Yang, Kim & Hwang (2012) reviewed the MRI findings of 97 patients with congenital superior oblique palsy, where an absent trochlear nerve with varying degrees of superior oblique muscle hypoplasia was documented in 73%. In comparison to those USOUA cases with a trochlear nerve and normal SOM characteristic on MRI, the former group presented at an earlier age with a prominent CHP and facial asymmetry. Although none of our children underwent specific MRI imaging of their SOM or trochlear nerve, each presented as infants/toddlers with the CHP being a particularly prominent feature and with a considerably lax SOT documented during surgery, a large incomitant vertical deviation ranging from 25 to 30 PDs, and various combinations of ipsilateral SOUA, ranging from –3 to –4 on versions and IOOA between +2 to +4 on versions (Vivian & Morris 1993).

A hallmark of this ‘early onset severe torticollis’ USOUA subgroup in this study was marked SOT laxity without increased inferior oblique muscle tone and macroscopically abnormal superior oblique tendons in a majority (group A; cases 1, 2 & 5). Unsurprisingly, each superior oblique surgical strengthening procedure was per-operatively individualised so as to achieve a normal to slight increase in superior oblique tone post-operatively.

This series, albeit small, provided historical evidence that children managed with single IOM weakening as their initial operation (cases 1, 2 & 5) had a poor outcome for torticollis in particular but also for their vertical tropia. In two (cases 1 & 2), there was little to no observable change to the torticollis even in the immediate post-operative period and an un-sustained partial reduction in the observed vertical tropia. In case 5, there was some temporary improvement in both, only to noticeably regress after circa 4 months. This contrasted with the authors post-surgery experience after combined and/or subsequent sequential secondary superior oblique surgery, which was supported by parental observation. These children achieved a persistent reduction or near-elimination of both torticollis and the symptomatic vertical tropia in 80% of cases. Enhancing the function of the SOT through varying resection and antero-transposition whilst weakening the antagonist IOM yielded stable outcomes and resolution or symptomatic reduction of the marked torticollis.

The finding that isolated weakening of the antagonist IOM was largely ineffective in reducing the excyclotorsion associated with these children’s torticollis/head-tilt, contrasted with the success of primary IOM weakening when correcting symptomatic vertical diplopia present in older children and adults (Coyle & MacEwan 2013; Kaeser, Klainguti & Kolling 2012; Raoof & Burke 2016). Indeed, Kaeser, Klainguti & Kolling (2012) concluded that inferior oblique recession (IOR) alone was very successful for managing congenital superior oblique palsy in adults (mean age 36 years), but a combined inferior oblique recession and superior oblique tuck should be considered in adults when the vertical deviation was particularly large in downgaze. Arici and Oguz (2012) found a superior oblique muscle tuck superior to any inferior oblique muscle procedure in older patients in reducing excyclotorsion. They did not investigate the response in young children.

We have described a challenging, well-recognised sub-group of young children with USOUA accompanied by severe ocular motility related torticollis, where the precise operative approach was influenced by the per-operative findings. We found that combining superior with inferior oblique muscle surgery achieved a marked improvement in the torticollis and in the vertical misalignment. Appropriate SO strengthening, which can be technically challenging and based on per-operative SOTT findings, appeared necessary to reduce the torticollis when its ipsilateral antagonist the variably overacting IO muscle is weakened, so as to maintain post-operative superior oblique function optimal. Even then, residual underaction of the SOM can persist and result in varying amounts of residual downgaze hyper phoria/tropia, which in this small series was confined to those cases with macroscopically documented abnormal SOTs. The residual incomitant downgaze hypertropia may merit additional surgical/non-surgical management in the longer-term (Kekunnaya & Isenberg 2014). Lau et al. (2009) reported that early surgery in their series was associated with a better outcome. The single case in this study who continued to have a symptomatic downgaze hypertropia and variable moderate torticollis was the oldest at both initial and subsequent surgery at 63 and 78 months, respectively, with a morphologically anomalous/very lax SOT. Does the presence of macroscopically anomalous superior oblique tendons and/or older age at initial superior oblique surgery contribute to less successful outcomes amongst this particular Congenital USOUA sub-group?

This study is limited by its small size, its retrospective nature and the need to finalise the amounts of superior oblique strengthening on the basis of the per-operative findings, clinical judgement and experience. Nonetheless, its findings, and those of the other small published studies, have highlighted the clinically and managerially distinct nature of this functionally and psychosocially disabling presentation of unilateral superior oblique muscle underaction, where the benefits of a larger prospective study cohort, perhaps through a collaborative multi-unit research study could better define its incidence and outcome spectrum, and lead to additional recommendations for both the optimum age at and best surgical practice.
